# Development and Evaluation of In-House ELISAs for the Detection of SARS-CoV-2-Specific Antibodies in COVID-19 Patients in Sri Lanka

**DOI:** 10.1155/2024/1331067

**Published:** 2024-09-19

**Authors:** Sisira L. Pathirana, Bhagya Deepachandi, Peshala Gunasekara, Narmada Fernando, Inoka C. Perera, Dakshika Gangani, James Thambyarajah, Dhanushka Dasanayake, Rajiva de Silva, Sunil Premawansa, Andreas Nitsche, Shiroma M. Handunnetti

**Affiliations:** ^1^ Institute of Biochemistry Molecular Biology and Biotechnology University of Colombo, Colombo, Sri Lanka; ^2^ Department of Life Sciences Faculty of Science, NSBM Green University, Mahenwaththa, Pitipana, Homagama, Sri Lanka; ^3^ Department of Zoology and Environment Sciences Faculty of Science University of Colombo, Colombo, Sri Lanka; ^4^ Department of Immunology Medical Research Institute, Colombo, Sri Lanka; ^5^ Center for Biological Threats and Special Pathogens Robert Koch Institute, Berlin, Germany

## Abstract

COVID-19 serological tests complement the molecular diagnostics and can be used as important tool for serosurveillance and vaccine efficiency evaluation. The aim of this study was to develop and evaluate the diagnostic performance of an in-house ELISA for retrospective serosurveillance of SARS-CoV-2. Total IgG and IgM levels in sera of PCR positive SARS-CoV-2 patients (*n* = 50) from North Colombo Teaching Hospital were evaluated and compared with sera (*n* = 50) collected from prepandemic healthy individuals as controls. Patient sample collection was initiated before vaccination programme was widely started within the country. Seropositivity of 94.0% (*n* = 47/50) was observed for either IgG or IgM anti-SARS-CoV-2 antibodies against receptor binding domain of spike protein or nucleocapsid protein in confirmed cases while none of controls were seropositive. In contrast, the seropositivity of only 48.0% (*n* = 24/50) was demonstrated with commercial ELISA kits for detection of IgG or IgM. All samples detected seropositive by commercially available kits remained seropositive with either in-house IgM or IgG ELISA. Significant correlations (*p* ≤ 0.001) were observed between Ab levels and day of sampling from the onset of illness. The overall sensitivity values of the in-house assays were 66.7%, 96.9%, and 100.0% for the first, second, and third week or longer after onset of symptoms for either in-house IgM or IgG ELISAs. Majority of the patients (>80.0%) were seropositive, regardless of age (<60 vs. >60 years), gender (male vs. female), or clinical severity (mild vs. moderate/severe). These data suggest that the developed in-house ELISAs can be applied to assess anti-SARS-CoV-2 antibody levels induced by either natural infections or vaccination.

## 1. Introduction

SARS-CoV-2 belongs to a large family of coronaviruses (CoVs), *Coronaviridae*, which are enveloped, single-stranded RNA viruses. There are four commonly circulating CoVs among humans (HCoVs), namely, HCoV2-NL63, -229E, -OC43, and -HKU1 [1]. The first three originated from bats, whereas the origin of HCoV2-HKU1 remains unknown. CoVs are capable of rapidly mutating giving rise to novel CoVs. Mutations of CoVs reported in 2002 leading to the occurrence of severe acute respiratory syndrome coronavirus (SARS-CoV) in China which is believed to have been transmitted from civet cats or bats to humans. A second novel CoV emerged in Saudi Arabia in 2012, named as Middle East Respiratory Syndrome Coronavirus (MERS-CoV) which is transmitted from dromedary camels to humans. The novel CoV (SARS-CoV-2) which was first detected in Wuhan City, the capital of Hubei province, China, in December 2019 showed genomic relatedness (87–89% nucleotide homology) to the bat SARS-related CoV found in Chinese horseshoe bats (bat-SL-CoVZC45) [2–4].

SARS-CoV-2, the coronavirus causing the COVID-19 pandemic, spread to many other countries within a few months causing a global pandemic. Cases of SARS-CoV-2 have been reported from 231 countries and territories around the world with more than 700,500,000 confirmed cases and more than 6,950,000 deaths reported by December 2023. Sri Lanka reported more than 672,500 confirmed cases and more than 16,800 deaths [5]. People with SARS-CoV-2 generally develop signs and symptoms in an average of 5-6 days after contracting the infection with a range of 1–14 days [6].

SARS-CoV-2 infection demonstrates a wide range of disease manifestations, asymptomatic, mild, severe-complicated, and fatal [7–9]. There is a high prevalence of asymptomatic or mild infections. However, they were undetectable with available diagnostic tests. Due to having a high prevalence of asymptomatic or mild infections, the true extent of virus spread within the population is underestimated, making containment of the infection and the decision making for the fight against SARS-CoV-2 difficult [10]. Therefore, rapid detection of the infection and the seroconversion status is important for decision making for control and containment of COVID-19.

Several types of tests are available for SARS-CoV-2 diagnosis including rapid antigen/antibody detection and real-time polymerase chain reaction (RT-PCR) [10]. However, commercially available diagnostic kits are expensive and at high demand, and sensitivity of some of the test kits is limited. Anti-SARS-CoV-2 antibodies developed either by natural infection or vaccination play a major role in mounting protection against SARS-CoV-2 infection. The development of an in-house antibody detection assay which is cost-effective and has higher sensitivity would thus be useful and important.

The current study developed and evaluated the diagnostic performance of in-house ELISAs and examined the serological response in SARS-CoV-2-infected patients in Sri Lanka. Furthermore, the usefulness of the developed assays for infection monitoring was described.

## 2. Methods

### 2.1. Study Participants and Sample Collection

Sample size required for the study was calculated using the standard formulae [11], TP + FN = *Z*^2^*X* [SN(1-SN)]/*W*^2^ and *n* = (TP + FN)/*P*, where TP: true positive, FN: false negative, SN: estimated sensitivity of ELISA, 98.0% (as determined based on literature published in other endemic settings [3, 12]), *Z*: normal distribution value at particular confidence interval (i.e., for 95%, *Z* = 1.96), *P*: seroprevalence in test population, 70.0% (as determined based on literature published in local setting [13, 14]), *W*: accuracy, 5.0%, and *n*: sample size.

Blood samples were collected from SARS-CoV-2 patients (*n* = 50), admitted at the North Colombo Teaching Hospital in 2020, whose infection was confirmed using RT-PCR. The clinical evaluation was carried out at the time of sample collection. None of the patients were vaccinated at the time of sample collection. Clinico-epidemiological data of the patients were recorded using a standard questionnaire. Healthy controls of 50 individuals whose serum samples were collected just before the pandemic started also were used as a negative control group. The sample collection was carried out by trained medical and paramedical personnel. Approximately 3 ml of venous blood was collected from each participant. The blood samples were incubated at room temperature for 30 minutes to 1 hour to allow clotting of blood. Then the serum was separated by centrifugation, aliquoted, and stored at −20°C until use.

### 2.2. Ethics Statement

Ethics approval for the study was obtained from the Ethics Review Committee of the Medical Research Institute, Ministry of Health, Sri Lanka (Ref. No. 24/2020). Written informed consent was obtained from each individual of patient and control groups prior to the collection of samples and data.

### 2.3. Selection of Antigen and Antibody

Commercially available products were used as coating antigen (Ag) and secondary antibodies (Abs) for the ELISA. SARS-CoV-2-specific recombinant proteins, i.e., recombinant human SARS-CoV-2 nucleocapsid (NC) his-tagged protein (cat no. ab273530 from Abcam) and recombinant human SARS-CoV-2 spike receptor binding (RBD) his-tagged protein (cat no. 10500-CV from Bio-Techne, R&D systems), were used as coating antigens while rabbit anti-human IgG H&L (HRP) (cat no. ab6759 from Abcam) and rabbit anti-human IgM *µ* chain (HRP) (cat no. ab97210 from Abcam) were used as secondary antibodies required for the ELISA.

### 2.4. Sequence Homology Analysis with Other Coronaviruses

Sequence homology analysis with other coronaviruses was carried out to determine the potential cross-reactivities. Closely related strains to SARS-CoV-2 were selected (i.e., SARS-CoV, MERS-CoV, hCoV-OC43, hCoV-NL63, hCoV-229E, and hCoV-HKU1), and relevant spike RBD and NC protein sequences were downloaded through NCBI (National Center for Biotechnology Information) or UniProt database. The sequences of SARS-CoV-2 spike and NC proteins were obtained through the details mentioned in the product sheets of commercial supplier (i.e., NCBI ID: YP_009724390.1 for spike protein and UniProt ID: P0DTC9 for NC protein). The relevant spike and NC protein sequences of SARS-CoV, MERS-CoV, hCoV-OC43, hCoV-NL63, hCoV-229E, and hCoV-HKU1 were obtained through protein ID P59594, W6A028, P36334, Q6Q1S2, P15423, Q0ZME7 and P59595, R9UM87, P33469, Q6Q1R8, P15130-1, Q5MQC6 respectively. Multiple sequence alignment for selected sequences was carried out using MUSCLE (MUltiple Sequence Comparison by Log-Expectation) tool (https://www.ebi.ac.uk/Tools/msa/muscle/) freely accessible through EMBL's (European Molecular Biology Laboratory's) European Bioinformatics Institute (EMBL-EBI). Heat map was further generated through MORPHEUS, the versatile matrix visualization and analysis software published by Broad Institute (https://software.broadinstitute.org/morpheus) [3].

### 2.5. Development of In-House ELISAs for Detection of SARS-CoV-2-Specific Antibodies

Prevalidation of the in-house ELISA was carried out using the standard checkerboard titration methods [15, 16]. ELISA was conducted with the optimized concentrations of each component at optimized reaction conditions. In brief, a 96-well ELISA plate was coated with either NC or RBD recombinant proteins in parallel containing at least 1 *µ*g/100 *µ*l of protein per well. The plate was incubated overnight at 4°C. Following overnight incubation, the plate was washed six times with wash buffer consisting of 1 X PBS supplemented with 0.2% Tween 20 (PBST). Plates were treated with 300 *µ*l/well of blocking buffer consisting of 1 X PBS with 5.0% non-fat milk. The plate was incubated at 37°C for 2 hours and washed again with PBST for six times. The plate was quickly dried at 37°C for 15–30 minutes and incubated with human serum samples at 1 : 100 dilution (100 *µ*l/well) for 1 hour at 37°C and washed six times with PBST. Secondary antibodies were added at 1 : 8000 dilution and at 100 *µ*l/well, and then the plates were incubated at 37°C for 30 minutes followed by washing with PBST for six times. TMB substrate (100 *µ*l/well) solution was used as detecting solution. The TMB containing plate was incubated 15 minutes at room temperature in the dark followed by stopping the reaction with 1 N HCl (50 *µ*l/well). ELISA reading was taken by measuring optical density values at 450 nm using Synergy HTX multimode microplate reader (BioTek, USA).

### 2.6. Validation of the In-House ELISAs

Validation of the in-house ELISA was carried out according to approved guidelines described on fundamental validation parameters for immunoassays which were presented in U.S. Pharmacopeia Chapter 1225, Validation on Compendial Methods, 2009, and ICH Q2 (R1) on Validation of Analytical Procedures: Text and Methodology, 2005 [17]. Cutoff values for new assays were determined according to the equation, *M*_healthy_ + 3SD_healthy_. Sensitivity, specificity, and positive (PPV) and negative (NPV) predictive values of the assays were further calculated using the 2 × 2 table analysis.

### 2.7. Quality Control

ELISA data were evaluated following established protocols [16]. In brief, each sample was analysed in duplicates and the mean value of absorbance was considered as the final value. Only absorbance values closer up to the second decimal point in duplicates were considered for calculating the mean. Each ELISA plate was run with an air blank, five or more healthy controls, and controls with and without conjugate. Normalization of day-to-day variations of the assay and test reproducibility were assessed according to acceptance and rejection criteria defined using mean absorbance values of healthy controls (*M*_healthy_), i.e., the mean absorbance value of healthy controls in each ELISA run should be within the range of *M*_healthy_ ± 2SDs. If more than four healthy controls fell outside the range, the test was rejected and repeated.

### 2.8. Assessment of SARS-CoV-2-Specific Antibodies Using EUROIMMUN ELISA Kits

Results obtained for in-house ELISAs were further compared with the commercially available RBD and NC protein-based ELISA kits for detection of anti-SARS-CoV-2 IgG and IgM antibodies supplied by EUROIMMUN, Germany. Each assay was carried out according to the manufacturer guidelines. Positive and negative controls (100 *µ*l of each) were initially added to the Ag coated ELISA plates according to the plate plan. The plate was incubated for 60 minutes at 37°C. Subsequently, the plate was washed three times using 300–450 *µ*l of working-strength wash buffer. Respective enzyme conjugate (secondary antibody) was added at 100 *µ*l per well and incubated for 30 minutes at 37°C. Washing step was repeated as in the previous step. The plate was further incubated for 30 minutes at room temperature with TMB substrate solution (100 *µ*l per well), and the reaction was stopped by adding 100 *µ*l of stopping solution. Absorbance measurements were obtained using an ELISA plate reader at 450 nm.

### 2.9. Comparison with Clinico-Epidemiological Data of Patients

Sensitivity of the developed assays was further analysed for detection of anti-SARS-CoV-2 antibodies in natural infection at different sampling times during the infection. Accordingly, the number of days from onset of symptoms in each patient with natural infection was recorded. Correlations were determined by linear regression analysis. Also, correlations of seropositivity with demographic and clinical characteristics of patients, i.e., age (<60 years vs. >60 years), gender (male vs. female), and clinical severity of the disease (mild vs. moderate/severe), were further analysed using SPSS version 25.0.

## 3. Results

### 3.1. Cross-Reactivity of SARS-CoV-2 with Other Coronaviruses

**Figure 1:**
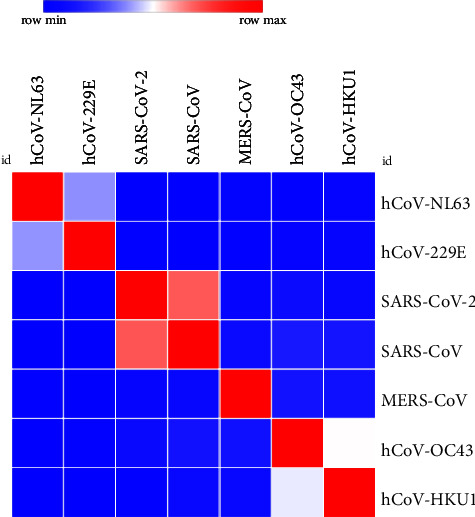
Analysis of heat map generated for RBD protein sequence used in the study to estimate relatedness to different strains of coronaviruses.

**Figure 2:**
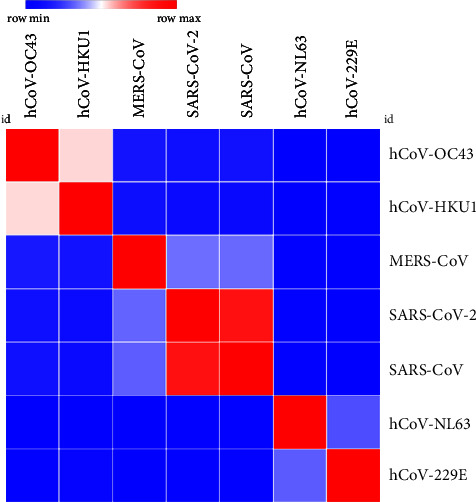
Analysis of heat map generated for NC protein sequence used in the study to estimate relatedness to different strains of coronaviruses.

According to sequence identity, only SARS-CoV demonstrated 73.06% (Table 1 and Figure 1) and 89.74% (Table 2 and Figure 2) identity to SARS-CoV-2 in spike RBD and NC protein sequences, respectively, while other analysed coronavirus variants demonstrated <50.0% identity to respective sequences of SARS-CoV-2.

### 3.2. Validation of the In-House ELISAs

**Figure 3:**
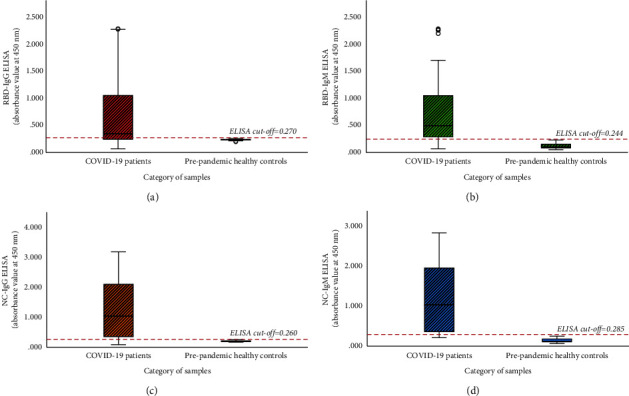
Comparison of ELISA values between COVID-19-infected patients and prepandemic healthy controls in (a) RBD-IgG ELISA, (b) RBD-IgM ELISA, (c) NC-IgG ELISA, and (d) NC-IgM ELISA. Boxes represent the interquartile interval, where 50% of the data was found.

According to the acceptance and rejection criteria used for evaluating inter-assay variations, the reproducibility of the ELISA was 100%. There was no assay repeated according to above criteria. Cutoff values were determined as 0.270, 0.244, 0.260, and 0.285 for RBD-IgG, RBD-IgM, NC-IgG, and NC-IgM ELISAs, respectively. None of the prepandemic healthy controls showed cross-reactivity in the developed ELISAs (Figure 3). As shown in Table 3, individual ELISAs for RBD demonstrated sensitivity from 70.0% to 84.0% while NC ELISAs demonstrated 82.0% to 88.0% sensitivity. When considering combined IgG and IgM ELISA results, RBD ELISAs demonstrated 84.0% sensitivity while it was 92.0% for NC ELISAs. Specificity and PPV remained 100.0% in all ELISAs while NPVs were >75.0% in each assay.

**Figure 4:**
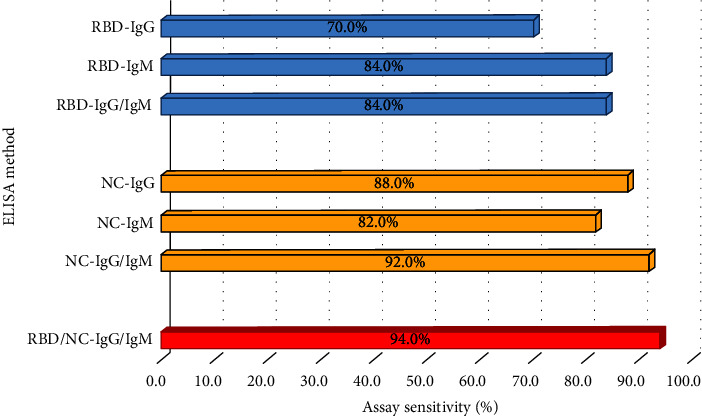
Variation of assay performance parameters in four types of individual ELISAs performed and when considered in combination.

As shown in Table 4, sensitivity was comparatively increased to 94.0% and specificity and PPV remained unchanged at 100.0%. Furthermore, NPV was 94.3% which was higher than individual ELISAs analysed in Table 3.  Figure 4 provides a summary of the comparison of assay sensitivities when considering IgG and IgM ELISAs with the two antigens RBD and NC separately and in combination.

In contrast, the seropositivity of only 48.0% (*n* = 24/50) was demonstrated with commercial ELISA kits for detection of IgG or IgM using either RBD or NC proteins while 28.0% (*n* = 14/50) demonstrated equivocal results and 24.0% (*n* = 12/50) demonstrated negative results. Furthermore, the commercial kit showed equal results for each assay (i.e., RBD-IgG, RBD-IgM, NC-IgG, and NC-IgM ELISAs).

In comparison to the commercial ELISA kits, the in-house assays demonstrated higher sensitivities for detection of serum antibodies in COVID-19 confirmed cases where 70.0% (*n* = 35/50), 84.0% (*n* = 42/50), 88.0% (*n* = 44/50), and 82.0% (*n* = 41/50) were detected as seropositive by RBD-IgG, RBD-IgM, NC-IgG, and NC-IgM assays, respectively (Table 3). Some samples which were negative or equivocal by the commercial assays were detected as positive in the in-house assays as shown in Table 5.

### 3.3. Comparison of In-House ELISA Results with Clinico-Epidemiological Data of Patients

The sensitivity of IgM and IgG ELISAs ranged between 33.3–50.0% and 33.3–66.7%, respectively, during the fihscrst week of postsymptom onset and was increased to 81.3–87.5% (for IgM) and 68.8%–90.6% (for IgG) by the second week. IgG ELISAs showed an approximately same sensitivity range (66.7%–83.3%) by third week while IgM ELISAs showed 100.0% sensitivity. When considering results of both IgM and IgG ELISAs for analysing sera of naturally infected patients, the sensitivity of the assay was 66.7%, 96.9%, and 100.0% for the first, second, and more than three weeks of postsymptom onset, respectively (Table 6). Antibody levels were statistically significantly and positively correlated (*p* ≤ 0.001) with the duration from onset of symptoms to the day of collection of the sample. This observation further confirms that the detection level of serological assays is dependent on the duration from the onset of illness to the day of sample collection.

Irrespective of age group (<60 years or >60 years), gender, or clinical severity (mild/moderate or severe), >80% of the patients were seropositive. There were no significant associations between seropositivity and age, gender, or clinical severity.

## 4. Discussion

This study describes the development of in-house ELISAs for detection of anti-SARS-CoV-2 antibodies in Sri Lankan patients [18]. Usually, the sensitivity of ≥90.0% is recommended for establishing a diagnostic assay for detection of symptomatic patients [19]; the described in-house ELISAs detected anti-SARS-CoV-2 antibodies in local patients with high sensitivity and specificity. There are no such methods developed locally for detection of serum antibodies against RBD or NC proteins in local patients. However, there were few studies conducted so far on detecting serum antibodies in naturally infected individuals and vaccinated individuals using established serological assays or in-house ELISAs based on different protein sequences of SARS-CoV-2 and for detection of neutralizing antibodies of the patients which will be useful in supporting vaccine developments and aiding in convalescent plasma therapy [13, 14, 20]. However, those antibody assays conducted using samples of naturally infected individuals demonstrated comparatively low assay performance.

Performance of serological assays depends on various factors such as assay design, antigenicity of viral epitopes, heterogeneity of antigens used, and secondary antibody isotypes used [12, 21]. Choosing the best antigen or antibody needs to be done carefully. The antigens can be used alone or in combination [3]. In this study, we used RBD and NC recombinant proteins as coating antigen of ELISA. Out of the four main structural proteins of coronaviruses (i.e., envelope (E), membrane (M), spike (S), and nucleocapsid (NC) proteins) [3], the most commonly used antigens for immunoassays are S1 with receptor binding domain (RBD) and the NC protein [9]. Most of the serological assays reported so far used complete S protein or S1/S2 subunits or the RBD with high sensitivity and specificity [3]. Serological tests allow for detection of antibodies from one to several weeks/months after infection or vaccination [22].

Results of the current study indicated that the detection level of serological assays is dependent on the sampling time from the onset of symptoms. As reported in the literature, seroconversion of IgM and IgG total antibodies in SARS-CoV-2-infected patients observed as early as one week from symptom onset and sensitivity of assays varied among 20.0–30.0% and 40.0–60.0% for IgM and IgG, respectively [3]. Considering individual antibodies, IgG assays remained low sensitive compared to IgM assays in initial three weeks from symptom onset [3]. The key time point of the disease process has been indicated as after 10 days from the symptom onset since most infected individuals produce antibodies during this period [23]. The findings of the current study are consistent with those findings. In the present study, sensitivity of IgM and IgG assays varied among 33.3–50.0% and 33.3–66.7%, respectively, during the first week from symptom onset and both IgG and IgM assays were 100.0% sensitive after three weeks of postsymptom onset.

Use of different sample types (e.g., saliva), different assays (e.g., T-cell assays and antigen detection assays), and different antibody classes (e.g., IgA) will give varying levels of sensitivity in serological assays at early stage of symptom onset, providing the opportunity for enhancing the sensitivity [18]. As reported recently, IgA and IgM are the earliest developed antibodies following a natural infection while IgG is formed later [22]. However, IgG denotes a higher specificity and guarantees a long-term protection than IgM [22]. Due to significant variability of antibody expression patterns in an infection, use of both IgM and IgG simultaneously is recommended for serological assays [12, 24]. Analysis of ELISA data in the current study also showed that the sensitivity was enhanced when both IgG and IgM assay results are combined. In our study, only 66.7% and 96.9% of patients showed seropositivity during the first and second weeks of symptom onset while all the patients showed seropositivity after the second week of symptom onset. Previous studies also demonstrated that total antibodies considering IgM and IgG together show high level of accuracy after the second week of infection, peaking at 2-3 weeks [2, 19, 21, 23–29]. Further follow-up studies would be important for evaluating the variation of serum antibody levels over time. Also, further studies should be conducted for assessing the ability of developed in-house ELISAs in detection of asymptomatic cases which would be helpful for preventing or curtailing the epidemics or pandemics [3].

The in-house assays tested in this study were 100.0% specific for detection of anti-SARS-CoV-2 antibodies. Results of patients and vaccinated people were only compared with prepandemic healthy controls and not with sera from patients with other viral or corona infections. Therefore, further phylogenetic analysis was carried out to evaluate the possible cross-reactivities between strains which are closely related to SARS-CoV-2 as suggested by other studies [3]. For the selected region of RBD and NC proteins, no possible cross-reactivities were observed with the analysed coronavirus species except SARS-CoV. As reported by other studies, cross-reactivity is expected with SARS-CoV infections due to the closeness of phylogeny (i.e., SARS-CoV and SARS-CoV-2) and their protein sequence identity compared to other coronaviruses [3, 30, 31]. However, the probability is very unlikely since even in Chinese population, it has been estimated that only about 8090 cases were affected by SARS-CoV which represents a small fraction of the Chinese population [31]. The higher identity value of NC compared to RBD protein between SARS-CoV and SARS-CoV-2 demonstrates the possibility of cross-reactivities in each. As mentioned by Algaissi et al., S1 protein shows less cross-reactivity among different coronaviruses compared to full-length S protein [3]. RBD protein belongs to S1 protein region of SARS-CoV-2 which further confirms these observations.

The developed assays may be used to estimate the true extent of seroconversion levels that represent true transmission of the disease which will be important to understand the infection risk and fatality rates. This will be immensely helpful for guiding public health policies as well as disease control and surveillance activities. However, as recommended by the U.S. Food and Drug Administration (FDA), more research is needed to carefully evaluate the positivity or negativity of an antibody test by analysing different groups of people including people with a prior SARS-CoV-2 infection, people who received COVID-19 vaccinations, and people who are not vaccinated or partially vaccinated. Further studies are ongoing for assessing serum antibody levels in vaccinated individuals using the developed ELISAs.

## Figures and Tables

**Table 1:** Percent identity matrix created by Clustal 2.1 for RBD protein sequence used in the study to estimate relatedness to different strains of coronaviruses.

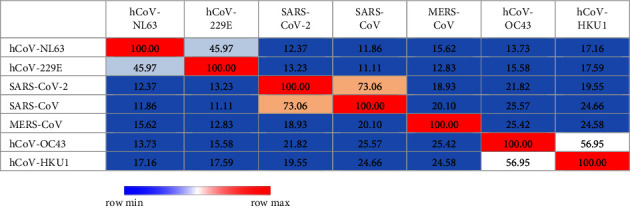

**Table 2:** Percent identity matrix created by Clustal 2.1 for NC protein sequence used in the study to estimate relatedness to different strains of coronaviruses.

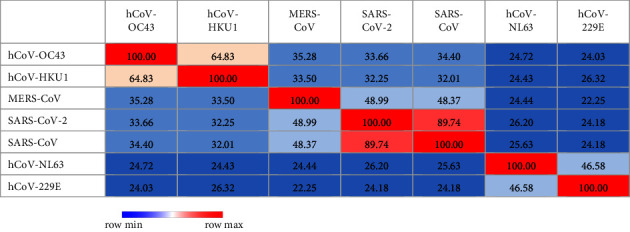

**Table 3:** Assay performance parameters of in-house ELISAs.

		Disease status defined by PCR		
		Positive	Negative	
RBD-IgG ELISA	Positive	35	0	PPV = 35/35 = 100%
	Negative	15	50	NPV = 50/65 = 76.9%
		SN = 35/50 = 70.0%	SP = 50/50 = 100%	

RBD-IgM ELISA	Positive	42	0	PPV = 42/42 = 100%
	Negative	8	50	NPV = 50/58 = 86.2%
		SN = 42/50 = 84.0%	SP = 50/50 = 100%	

RBD-IgG/IgM ELISA	Positive	42	0	PPV = 42/42 = 100%
	Negative	8	50	NPV = 50/58 = 86.2%
		SN = 42/50 = 84.0%	SP = 50/50 = 100%	

NC-IgG ELISA	Positive	44	0	PPV = 44/44 = 100%
	Negative	6	50	NPV = 50/56 = 89.3%
		SN = 44/50 = 88.0%	SP = 50/50 = 100%	

NC-IgM ELISA	Positive	41	0	PPV = 41/41 = 100%
	Negative	9	50	NPV = 50/59 = 84.7%
		SN = 41/50 = 82.0%	SP = 50/50 = 100%	

NC-IgG/IgM ELISA	Positive	46	0	PPV = 46/46 = 100%
	Negative	4	50	NPV = 50/54 = 92.6%
		SN = 46/50 = 92.0%	SP = 50/50 = 100%	

SN: sensitivity, SP: specificity, PPV: positive predictive value, and NPV: negative predictive value.

**Table 4:** Combined RBD and NC ELISAs.

		Disease status defined by PCR		
		Positive	Negative	
Combined RBD/NC ELISAs	Positive	47	0	PPV = 47/47 = 100.0%
	Negative	3	50	NPV = 50/53 = 94.3%
		SN = 47/50 = 94.0%	SP = 50/50 = 100.0%	

**Table 5:** Comparison of assay performances of commercial and in-house ELISAs.

			Seropositivity by in-house ELISA			
			RBD-IgG ELISA	RBD-IgM ELISA	NC-IgG ELISA	NC-IgM ELISA
Total study group			70.0% (*n* = 35/50)	84.0% (*n* = 42/50)	88.0% (*n* = 44/50)	82.0% (*n* = 41/50)
Commercial ELISA kits (RBD-IgG, RBD-IgM, NC-IgG, or NC-IgM)	Seropositives	48.0% (*n* = 24/50)	100.0% (*n* = 24/24)	100.0% (*n* = 24/24)	100.0% (*n* = 24/24)	95.8% (*n* = 23/24)
	Equivocal	28.0% (*n* = 14/50)	71.4% (*n* = 10/14)	92.9% (*n* = 13/14)	92.9% (*n* = 13/14)	85.7% (*n* = 12/14)
	Seronegatives	24.0% (*n* = 12/50)	8.3% (*n* = 1/12)	41.7% (*n* = 5/12)	58.3% (*n* = 7/12)	50.0% (*n* = 6/12)

**Table 6:** Serum antibody levels in natural infection evaluated by the described invention.

		Sensitivity (%) by considering date of sample collection (number of dates after symptom onset)			
In-house ELISAs (based on two different recombinant antigens used)	Specificity (%)	1^st^ week (1 to 7 days) (*n* = 6/50)	2^nd^ week (8 to 14 days) (*n* = 32/50)	3^rd^ week (15 to 21 days) (*n* = 6/50)	≥4^th^ week (>21 days) (*n* = 6/50)
RBD-IgG ELISA	100.0	33.3	68.8	66.7	100.0
RBD-IgM ELISA	100.0	33.3	87.5	100.0	100.0
NC-IgG ELISA	100.0	66.7	90.6	83.3	100.0
NC-IgM ELISA	100.0	50.0	81.3	100.0	100.0
Combined RBD/NC-ELISA	—	66.7	96.9	100.0	100.0

## Data Availability

The data supporting the conclusions of this article are included within the article. Other data have not been made available as they were not part of the ethics application and due to patient confidentiality.
